# Gene-Swapping Mediates Host Specificity among Symbiotic Bacteria in a Beneficial Symbiosis

**DOI:** 10.1371/journal.pone.0101691

**Published:** 2014-07-11

**Authors:** Alba A. Chavez-Dozal, Clayton Gorman, C. Phoebe Lostroh, Michele K. Nishiguchi

**Affiliations:** 1 New Mexico State University, Department of Biology, Las Cruces, New Mexico, United States of America; 2 Colorado College, Department of Biology, Colorado Springs, Colorado, United States of America; Institut Pasteur, France

## Abstract

Environmentally acquired beneficial associations are comprised of a wide variety of symbiotic species that vary both genetically and phenotypically, and therefore have differential colonization abilities, even when symbionts are of the same species. Strain variation is common among conspecific hosts, where subtle differences can lead to competitive exclusion between closely related strains. One example where symbiont specificity is observed is in the sepiolid squid-*Vibrio* mutualism, where competitive dominance exists among *V. fischeri* isolates due to subtle genetic differences between strains. Although key symbiotic loci are responsible for the establishment of this association, the genetic mechanisms that dictate strain specificity are not fully understood. We examined several symbiotic loci (*lux*-bioluminescence, *pil* = pili, and *msh*-mannose sensitive hemagglutinin) from mutualistic *V. fischeri* strains isolated from two geographically distinct squid host species (*Euprymna tasmanica*-Australia and *E. scolopes*-Hawaii) to determine whether slight genetic differences regulated host specificity. Through colonization studies performed in naïve squid hatchlings from both hosts, we found that all loci examined are important for specificity and host recognition. Complementation of null mutations in non-native *V. fischeri* with loci from the native *V. fischeri* caused a gain in fitness, resulting in competitive dominance in the non-native host. The competitive ability of these symbiotic loci depended upon the locus tested and the specific squid species in which colonization was measured. Our results demonstrate that multiple bacterial genetic elements can determine *V. fischeri* strain specificity between two closely related squid hosts, indicating how important genetic variation is for regulating conspecific beneficial interactions that are acquired from the environment.

## Introduction

Environmentally transmitted symbioses occur through the acquisition of bacteria from the environment into a naïve, un-colonized juvenile host [1]. This type of transmission strategy can be complex, since bacteria are obtained anew for each generation of hosts, and is dependent upon the population and type of symbionts present when transmission occurs [2], [3]. Both host and environment have strong influences upon symbiont fitness, and it is the interplay between these two forces that determine whether specific symbiotic strains are able to colonize and persist generation after generation [4], [5]. One example where both host and environment exert notable selection pressures upon symbiotic bacteria is in the sepiolid squid-*Vibrio* mutualism [5].

Complex molecular dialogs (including genetic interdependence) exist between sepiolid squid hosts and their *Vibrio* bacteria, leading to a highly specific association and subsequent cospeciation [5]–[8]. The complex processes by which hosts and symbionts find each other (among the tremendous marine bacterial community) in order to initiate a successful mutualism include a myriad of well-defined molecular signaling events that dictate a certain “conversation” between partners [9]. Additionally, it has been reported that both bacterial specificity (where Vibrios preferentially colonize particular species of hosts as well as environment [6], [10], [11] dictate which symbionts are successful in squid light organ symbioses. Studies competing native strains with non-native strains in both allopatric Australian *Euprymna tasmanica* and Hawaiian *Euprymna scolopes* indicate the existence of competitive dominance and intraspecific recognition of environmentally transferred symbionts [6], [8], [11]. Along with host specificity, environmental temperature is also an important factor for colonization and dominance of specific *Vibrio* strains when colonizing different squid host species living in sympatry [4]. Thus, bacterial specificity is dictated both by host mechanisms of selection (particular *Vibrio* spp. are host specialists) or the environment (vibrio bacteria as a group are host generalists), but the exact means of this specificity have not been determined [5].

Recent studies have been devoted to defining bacterial mechanisms (for example gene activation, horizontally transmitted elements, mutations, duplications, *etc*.) for host specificity. Recenty, a study demonstrated that, under laboratory conditions, host specificity between sepiolid squids and one species of monocentrid fish was determined by the presence of a single gene in *V. fischeri* (*rscS*), which regulates luminescence and synthesis of the symbiosis polysaccharide locus (*syp*) that is important for host colonization and biofilm formation [7]. What subtle genetic factors are responsible for the dramatic competitive fitness differences between various isolates of *V. fischeri* among all sepiolid squids, rather than between squids and a completely different vertebrate host, is the focus of this study.

Two closely related *V. fischeri* isolates (ETJB1H from the Australian host *Euprymna tasmanica*, and ES114 from the Hawaiian host *E. scolopes*) were examined in order to determine whether differences in symbiotic loci were important for strain specificity and host recognition. These two *V. fischeri* strains were selected because they can each colonize aposymbiotic hatchlings from both species of squid equally well in 48 hour colonization assays so long as they are the only strain of *V. fischeri* present; and they nevertheless also demonstrate competitive dominance when they are presented together to their native host. That is to say Australian *E. tasmanica* hosts are preferentially colonized by *V. fischeri* ETJBH1 when *V. fischeri* ETJBH1 and *V. fischeri* ES114 bacteria are both present, and *E. scolopes* are preferentially colonized by *V. fischeri* ES114 when *V. fischeri* ETJBH1 bacteria are also present [6], [8], [11]. It is likely that subtle differences in specific symbiotic loci are responsible for this complex phenotype. The two particular bacterial strains were also selected because they have full or partial sequenced genomes, allowing easy genetic comparisons between both strains [10].

## Materials and Methods

This study was carried out in strict accordance with the recommendations in the Guide for the Care and Use of Laboratory Animals of the National Institutes of Health. The protocol was approved by the Institutional Biosafety Committee of New Mexico State University (Permit Number: 1306NMD20103) and under the guidelines of the NMSU's Institutional Animal Care and Use Committee (85-R-009 and OLAW A4022-01 and IACUC license 2013-029). Animals were appropriately handled with care and under appropriate conditions to minimize any suffering [5]. Adult *Euprymna tasmanica* were collected from Botany Bay, New South Wales, Australia with permits from the Australian Government, Department of Sustainability, Environment, Water, Population, and Communities (Export permit WT2013-10343), the New South Wales Government, Industry and Investment (Collection permit P04/0014-6.0), and the Australian Government Department of Agriculture, Fisheries, and Forestry Biosecurity (AQIS invoice ELS0016507329). *Euprymna scolopes* (Kane'ohe Bay, Honolulu, O'ahu, GPS coordinates- N 21°26′ W 157°47′) were not required to have any collection permits at this site. Both species of *Euprymna* are not endangered or are protected in either location.

### Bacterial strains and growth conditions

Two *V. fischeri* strains were chosen for this study: *V. fischeri* ETJB1H isolated from the light organ of *Euprymna tasmanica* from Jervis Bay, Australia and *V. fischeri* ES114 isolated from the light organ of *Euprymna scolopes* from Kane'ohe Bay, Hawaii. Both strains were grown in Luria Bertani high Salt (LBS; per liter composition: 10 g tryptone, 5 g yeast extract, 20 g NaCl, 50 mL 1 M Tris pH 7.5, 3.75 mL 80% glycerol and 950 mL dH_2_O) media and shaken at 225 rpm at 28°C overnight.

### Mutant construction

#### Campbell mutations

Luciferase (*lux*) mutants of both strains (*V. fischeri* ETJB1H and ES114) were constructed by insertion of the plasmid pEVS122 as described previously (11) and constructs are listed in Table S2. Briefly, the *luxA* gene was partially amplified with specific primers designed from the sequenced strain *V. fischeri* ES114 (NCBI accession: NC_006840.2). PCR products were purified and cloned (after double digestion of PCR products and plasmid with *Xba*I and *Xma*I, with posterior ligation in a 1∶3 plasmid-insert ratio) into the suicide vector pEVS122, and wild type *V. fischeri* strains were transformed by tri-parental mating via conjugation through a helper strain [12]. Strains that had undergone single homologous recombination events with the native gene were selected on LBS plates enriched with erythromycin (25 µg/mL). Strains constructed were defined as ES114::pACH101 (for the *lux* mutant of the Hawaiian *V. fischeri* strain ES114) and ETJB1H::pACH102 (for the *lux* mutant of the Australian *V. fischeri* strain ETJB1H). Constructs were verified by Southern blotting.

#### Allelic exchange

*msh* and *pil* mutants (*mshINQ* and *pilABCD*) were constructed by allelic replacement of the chromosomal loci as described previously [13]. 500 bp of neighbor genes were amplified and cloned (the first insert was cloned after digestion of plasmid and PCR product with *Sma*I and *Bam*H1, with posterior ligation in a 1∶5 plasmid-insert ratio; the second insert was cloned after digestion of plasmid and PCR product with *Spe*I and *Xba*I, with posterior ligation in a 1∶10 plasmid-insert ratio) into the suicide vector pSW7848 containing the P_BAD_ promoter and a chloramphenicol resistant cassette (Tables S1, S2). After transformation of ultracompetent cells (NEB 10-beta competent *E. coli*, New England BioLabs, MA, USA), selection was achieved through antibiotic enrichment (5 µg/mL choloramphenicol). Recipient cells (*V. fischeri*) were transformed by tri-parental mating as described above. Transformed strains (with respective deletions) were selected through colony patching after inoculation in LBS media enriched with 2% arabinose to allow dismissal of inserted constructs through expression of the toxic gene *ccdB* by activation of the P_BAD_ promoter after incubation in LBS media with arabinose. To easily discriminate transformants, colonies from the original tri-parental mating that were initially resistant to chloramphenicol and eventually insensitive to CcdB toxicity were selected. Constructs were named by their respective deletion (Table S2) and were verified by PCR.

### Complement construction

#### GAPture or TAR cloning

Complementation of the *lux* operon with the opposite strain's loci (*lux* in ETJB1H for ES114::pACH101 and *lux* in ES114 for ETJB1H::pACH102) was achieved through operon mobilization (“TAR cloning” or “GAPture”) as previously described [14]. TAR cloning technique was achieved by using the yeast homologous recombination pathway. 700 bp of neighboring genes (upstream and downstream) from the *luxCDABEG* operon (with 40 nucleotides of the 5′ end that were homologus with vectors pCRG13 and pCRG23) were PCR amplified and purified. Vector pCRG23 was digested with *Srf*I and pCRG13 was digested with *Eco*RV. In *Saccharomyces cerevisiae* transformation, the yeast strain CRY1-2 (containing the genotype *ura* ^−^, *leu* ^−^, *cyh2^R^*, that confers sensitivity to cyclohexamide and cannot grow in media without uracil) was co- transformed with the two digested plasmids and the amplified upstream and downstream genes using lithium acetate transformation. After transformation, yeast colonies were plated on synthetic URA medium (per liter composition: adenine hemisulfate 0.18 g, arginine HCl 0.12 g, glutamic acid 0.6 g, histidine HCl 0.12 g, myo-inositol 0.2 g, isoleucine 0.18 g, leucine 0.18 g, lysine HCl 0.18 g, methionine 0.12 g, p-aminobenzoic acid 0.02 g, phenylalanine 0.3 g, homoserine 0.6 g, tryptophan 0.24 g, tyrosine 0.18 g, valine 0.9 g, Difco yeast nitrogen base without aminoacids 6.67 g, glucose 20 g) and incubated at 30°C for 4 days. After incubation, colonies were suspended *en masse* with 10 mL of TE and transferred into a 15 mL falcon tube, spun, and the construct (two plasmids + upstream/downstream genes) was extracted with glass beads and 200 µL of glass beading solution (per liter composition: 5 mL 20% sodium dodecil sulfate, 10 mL 1 M NaCl, 1 mL 1 M Tris-HCl pH 8.0, 1 mL 0.1 M EDTA, 2 mL Triton X100) and purified with Phenol/Chloroform. Plasmid DNA was transformed with ultracompetent *E. coli* cells (NEB 10-beta competent *E. coli*, New England BioLabs, MA, USA) and incubated for 24 hours at 37°C. Transformed cells (∼ 10 colonies) were re-inoculated and plasmid DNA was extracted using the Qiagen plasmid Maxi kit (QIAGEN Inc., CA, USA). For *lux* operon cloning step, CRY 1–2 yeast cells were transformed (CaCl_2_ spheroplast transformation procedure) with 1 µg of the plasmid extract and 5 µg of genomic DNA (from either *V. fischeri* ES114 or ETJB1H). Yeast cells were plated onto TYC1/Cycloheximide plates (per liter composition: D-sorbitol 182.2 g, Difco yeast nitrogen base without aminoacids 6.75 g, dextrose 0.98 g, adenine 0.2 g, arginine 0.2 g, aspartic acid 1 g, histidine 0.2 g, leucine 0.59 g, lysine 0.53 g, methionine 0.2 g, phenylalanine 0.4 g, threonine 2 g, tryptophan 0.2 g, tyrosine 0.3 g and 3 mg/mL cycloheximide) and incubated at 30°C for 7 days. Plasmids (containing the *lux* operon) were extracted from yeast spheroplasts using the Stratagene strataprep plasmid miniprep kit (Fisher Scientific, PA, USA). Constructs were verified by PCR and Southern blotting. *E. coli* ultracompetent cells were transformed and triparental mating was achieved as described previously. *V. fischeri* ES114::pACH101 was complemented with the *lux* operon of *V. fischeri* ETJB1H and strain *V. fischeri* ETJB1H::pACH102 was complemented with the *lux* operon of *V. fischeri* ES114.

#### Cloning using the conjugal vector pVSV105

Complete copies of the loci for *msh* and *pil* operons were amplified with specific primers for the entire locus (Table S1). PCR products and the vector pVSV105 [15] were double digested with *Xba*I and *Xma*I digests were ligated in a 1∶3 plasmid-insert ratio and transformed into ultracompetent *E. coli* cells (NEB 10-beta competent *E. coli*, New England BioLabs, MA, USA). Cells were selected with chloramphenicol enrichment (25 µg/mL) and *V. fischeri* recipient cells were transformed by tri-parental mating and selected after chloramphenicol enrichment (5 µg/mL). Lastly, we constructed complements that contain each strain's native loci by cloning the respective locus into vector pVSV105. Transformation was then performed as described previously (see Table S2 for complete list of complements). Complemented strains were verified by Southern blot.

### Colonization assays

Colonization assays were performed as described previously [16]. Overnight cultures of *V. fischeri* wild-type strains (ES114 and ETJB1H), mutants and complements were regrown in 5 mL of fresh LBS media until they reached an OD_600_ of 0.3. For single and competition infection experiments, cultures were then diluted to approximately 1×10^3^ CFU/mL in 5 mL of sterile artificial seawater and added to glass scintillation vials where newly hatched juvenile squids were placed (one individual/vial). Seawater was changed with fresh uninoculated artificial seawater every 12 hours over a period of 48 hours. Animals were maintained on a light/dark cycle of 12/12. After 48 hours, animals were sacrificed and homogenized, and the diluted homogenate was plated onto LBS agar plates for the wild-type *V. fischeri*, LBS with erythromycin (25 µg/mL) for the *V. fischeri* mutants, and LBS with chloramphenicol (5 µg/mL) for the *V. fischeri* complements. A second set of animals were selected for competition studies where juvenile squids were co-infected with the native strain and a respective complement (Table S2), sacrificed after 48 hours, homogenized, and plated onto the various media as reported above. Colony forming units (CFUs) were counted the next day to determine colonization efficiency of each strain. A total of 8 animals/strain were used for each competition assay, and 10 non-infected (aposymbiotic) juveniles were used as negative controls.

### Statistical analysis

To compare bacterial populations (wild-type, mutant, and complement constructs), one way ANOVA followed by the Tukey comparison was performed on calculated CFU numbers. Three technical replicates and 10 biological replicates (representing 3 treatments with 10 animals/strain and one set of 10 non-infected or aposymbiotic animals for the negative control).

## Results and Discussion

We disrupted loci from three operons that were previously reported to be important for host colonization and persistence: *lux* (light production), *msh* (biofilm formation) and *pil* (attachment to host). First, gene disruption was achieved via single recombinational events and allelic exchange. Secondly, complementation in *trans* of mutants with copies from the other strain was achieved by the *Saccharomyces cerevisiae*-based molecular tool (GAPture) for operon manipulation and mobilization (*lux*) and extrachromsomal maintenance (*pil* and *msh*). For detailed information of plasmids and strains constructed and used in this study, see supplementary data (Tables S1, S2). Finally, animal colonization experiments were performed using host-specific (or native) strains and complemented mutants in both squid host species (*E. tasmanica* or *E. scolopes*) to determine whether these loci were involved in strain recognition with the purpose of describing how competitive hierarchy is linked to the manipulated symbiotic operons. Using single and competitive colonization experiments for all strains constructed (Table S1), colonization studies were performed in naïve hatchlings of both *E. scolopes* and *E. tasmanica* animals (Figures S1–S3). Competition assays between the two wild type *V. fischeri* strains (ETJB1H and ES114) exhibit the expected host preference for the native strain, where native *V. fischeri* significantly out-competed non-native strains during colonization, supporting earlier work [5], [6].

**Figure 1:**
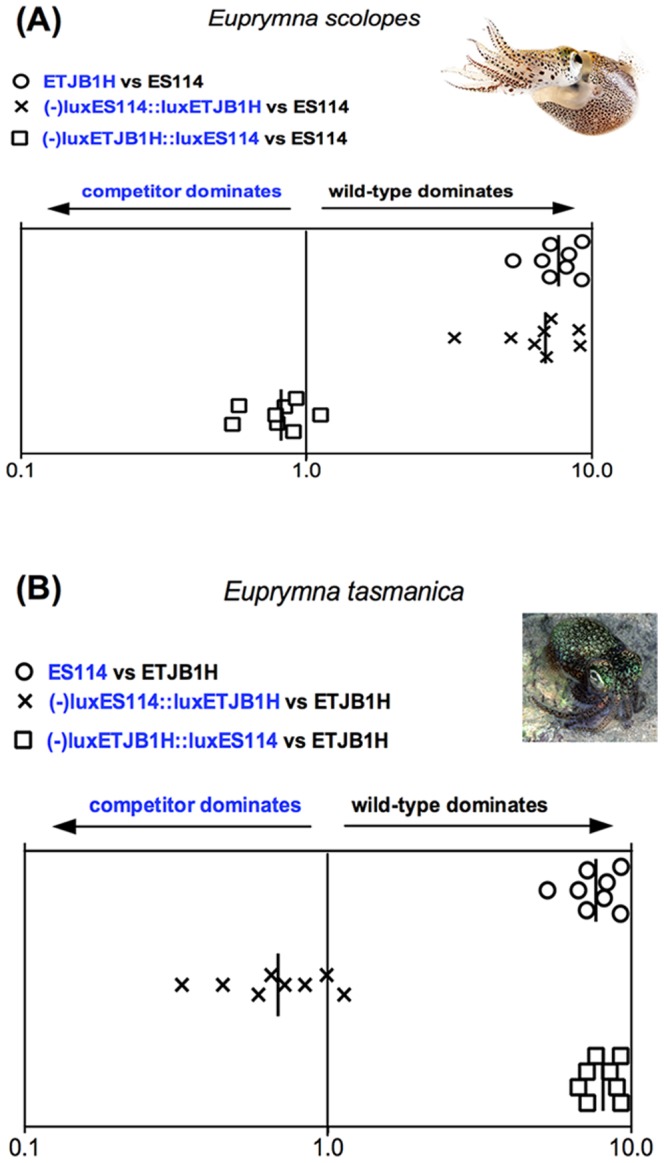
*lux* operon data. Colonization assays 48-hour post-infection of juvenile (A) *Euprymna scolopes* and (B) *Euprymna tasmanica* by their respective wild-type (ES114 or ETJB1H), mutant, and complement strains of the *lux* operon for *Vibrio fischeri*. Infection efficiency data is plotted as the log values of the relative competitiveness index (RCIs), calculated by dividing the ratio of mutant to wild-type by the starting ratio [28]. If the RCI is <1 the mutant strain was outcompeted by the wild-type, the wild-type strain was outcompeted by the mutant if the value is >1, and a RCI equal to 1 indicates no competitive difference. Data points represent individual animals and the position of the figures on the y axis is merely for spacing. Vertical line represents the median value of each data plot.

Infection studies in Hawaiian juvenile *E. scolopes* competed native Hawaiian *V. fischeri* ES114 against non-native Australian *lux- V. fischeri* ETJB1H strain complemented with either the native ES114 *lux* or the ETJB1H *lux* genes (Figs. 1, S1). The *lux*- non-native *V. fischeri* ETJB1H had equal competitive ability against native *V. fischeri* ES114 exclusively when its *lux* mutation had been complemented with native ES114 *lux* (Fig. 1). That is, *lux*- non-native ETJB1H behaved like ES114 exclusively when the *lux*- mutation was complemented with the ES114 *lux* operon. Furthermore, when the *lux*- native Hawaiian strain (ES114) was complemented with the non-native *lux* operon from the Australian strain (ETJB1H) and competed against native *V. fischeri* ES114, the wild type dominated the complemented strain (Fig. 1). That is to say the *lux*- ES114 strain complemented with the *lux* operon from non-native ETJB1H behaved like the non-cognate ETJB1H strain.

Results of similar experiments completed in *E. tasmanica* juveniles (where *V. fischeri* ETJB1H is native, and *V. fischeri* ES114 is non-native) produced the expected reciprocal results. For example, when *lux* from non-native Hawaiian *V. fischeri* (ES114) was replaced with native *lux* from Australian *V. fischeri* ETJB1H (strain –*lux*ES114::*lux*ETJB1H), there was an increase in colonization efficiency of the competitor as if using the native wild type Australian ETJB1H strain. Additionally when native Australian ETJB1H strain was mutated (-*lux*) and complemented with non-native Hawaiian ES114 *lux* (strain –*lux*ETJB1H::*lux*ES114), colonization efficiency was as if the native wild type Australian ETJB1H strain had been competed against itself (Figs. 1, S1). These results indicate that complementary *lux* genes are equally proficient at determining host preference in both *E. tasmanica* and *E. scolopes* squid hosts, indicating that phenotypic plasticity at one locus can give a subtle advantage to a non-native symbiont, even though it may not be the only gene responsible for symbiont recognition and specificity.

The *lux* operon is responsible for biosynthesis of luciferase, which has a crucial role in *V. fischeri* bioluminescence and fitness. Light production is used by the squid to avoid predation via silhouette reduction in a behavior known as counterillumination [16], [17]. The *lux* operon is present in *V. fischeri* as a conserved, contiguous, and coordinately expressed set of genes that have thought to have been acquired through horizontal gene transfer (HGT) among closely related bacterial clones and through vertical inheritance between bacterial families (*e.g*., *Vibrionaceae* and *Enterobacteriaceae*). Results from this part of our study indicate that although both *lux* operons produce bioluminescence and their structural proteins are similar in primary sequence, host specificity can be obtained through artificial HGT of the *lux* operon alone [18]. Our cloning method intentionally included 5′ and 3′ noncoding sequences flanking the operons; perhaps noncoding sequences contribute to the observed host preference. The *lux* operon might therefore drive evolutionary strain speciation through non-reproductive transmission of *lux* genes, when *lux* DNA is available in the environment and there are no other constraints on integration of operons into the recipient cell (e.g. the action of restriction endonucleases). Additionally, natural competence has been previously observed in *V. fischeri* after expression of the transcriptional regulators *tfoX* and *tfoY* (chitin-sensing regulators); this earlier study highlights a conserved mechanism of genetic exchange in the presence of chitin [19].

**Figure 2:**
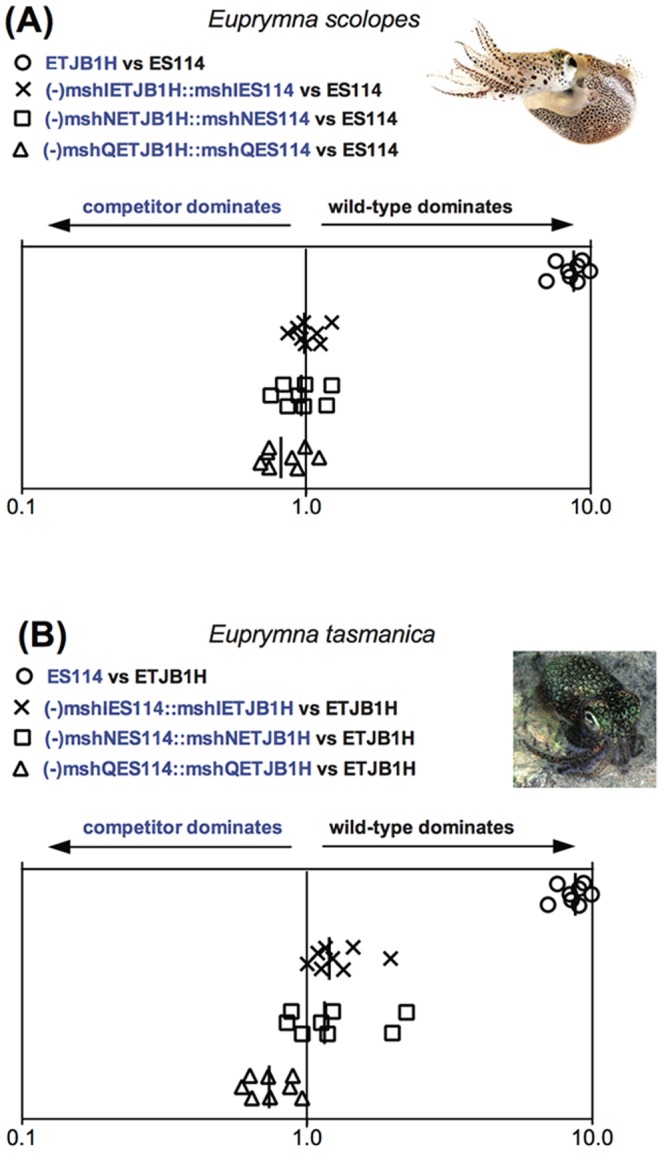
*msh* operon data. Colonization assays 48-hour post-infection of juvenile (A) *Euprymna scolopes* and (B) *Euprymna tasmanica* by their respective wild-type (ES114 or ETJB1H), mutant, and complement strains of *msh* genes for *Vibrio fischeri*. Infection efficiency data is plotted as the log values of the relative competitiveness index (RCIs), calculated by dividing the ratio of mutant to wild-type by the starting ratio [28]. If the RCI is <1 the mutant strain was outcompeted by the wild-type, the wild-type strain was outcompeted by the mutant if the value is >1, and a RCI equal to 1 indicates no competitive difference. Data points represent individual animals and the position of the figures on the y axis is merely for spacing. Vertical line represents the median value of each data plot.

Multiple genes comprise the entire *msh* operon (including *mshABCDGIJLMNOPQ*), which is responsible for the synthesis of type IV pseudopili, important for biofilm formation [20] and attachment (or adherence) to abiotic surfaces [21]. The *msh* operon has also been reported to be crucial for attachment to certain host tissues, which is an important step for successful colonization and persistence [11]. We specifically targeted *mshI, mshQ* and *mshN*, since these proteins exhibit high variability in their primary sequence among multiple strains of *V. fischeri*, including Hawaiian ES114 and Australian ETJB1H (C. Lostroh, unpublished data). Loci from the *msh* operon were mutated by means of insertional inactivation and complemented by extrachromosomal maintenance [13], [15], [22]. Similar to the *lux* operon experiments, ES114 mutant strains were complemented with the ETJB1H *msh* gene, and vice versa. Colonization tests using all mutant strains were then completed in both *E. tasmanica* and *E. scolopes* juvenile squids. Results of *mshI, mshN* and *mshQ* loci after colonization are illustrated in Figure 2. Due to the difficulty of obtaining a large number of animals from one clutch to complete all infection experiments with *msh* strains, we used three different clutches from *E. scolopes* and two from *E. tasmanica*. Inter-clutch colonization variability was observed between groups, and reflected in low numbers in competition experiments; however, animals from the same clutch were used to replicate the same competition experiment to avoid variation in colonization efficiency.

Mutation of the *msh* genes in Hawaiian *V. fischeri* ES114 caused a significant reduction in colonization efficiency in *E. scolopes* (data not shown), demonstrating that the *msh* operon is important for symbiotic competence. When Hawaiian *E. scolopes* were infected with non-native ETJB1H complemented with native *mshI* (-*mshI*ETJB1H::*mshI*ES114), colonization efficiency was equal to the native wild-type (ES114). Results were similar for *mshL* and *mshQ*. These observations indicate that *mshI*, *mshL*, or *mshQ* are all important in conferring host specificity between *E. scolopes* and *E. tasmanica* squids. Interestingly, colonization in *E. tasmanica* juveniles did not mirror these results as they did for the *lux* operon. Overall levels of colonization in this case were very low (Fig. S1). The *mshI* and *mshN* ES114 strains complemented with their ETJBH1 *msh* counterparts were out-competed by wild type ETJBH1, while the *mshQ* ES114 strain complemented with its ETJBH1 *msh* counterpart competed slightly better for colonization than wild type ETJBH1. Alternatively, ES114 complemented with native ETJB1H *mshQ* locus (-*mshQ*ES114::*mshQ*ETJB1H) outcompeted the wild-type strain. Thus, results for *mshQ* closely resemble those for *lux*, whereas *mshI* and *mshN* favored one *V. fischeri* strain (ETJB1H) but not the other (ES114). In our mixed competitions using *mshI* or *mshN* mutants, *E. tasmanica* hosts select against all complemented bacteria, keeping total levels of each symbiont low (Figs. 2, S2). In addition to clutch variability, *E. tasmanica* hosts may exert stronger sanctions against non-native *V. fischeri* more than its congener *E. scolopes* due to the presence of a genetically diverse group of *V. fischeri* symbionts available for colonization in the *E. tasmanica* habitat, whereas *V. fischeri* symbionts from *E. scolopes* are more homogeneous and host squids sample from only a small set of *V. fischeri* genotypes [5]. Having the ability to discern amongst a large, genetically diverse pool of *V. fischeri* may give squids an advantage to also differentiate cheaters to allow for a more successful beneficial symbiosis [23]. Recent work has demonstrated changes in particular symbiotic traits (luminescence, biofilm production, motility, carbon source utilization, growth) of Hawaiian *V. fischeri* strain ES114 when evolved in *E. tasmanica* hosts [24]. These traits differ quite dramatically, with the evolved strain gaining traits similar to the native strain over time. Thus, our results indicate that the *msh* operon is not only important for successful colonization of sepiolid squids, but also determines host range and accommodation from a large pool of available *Vibrio* symbionts.

**Figure 3:**
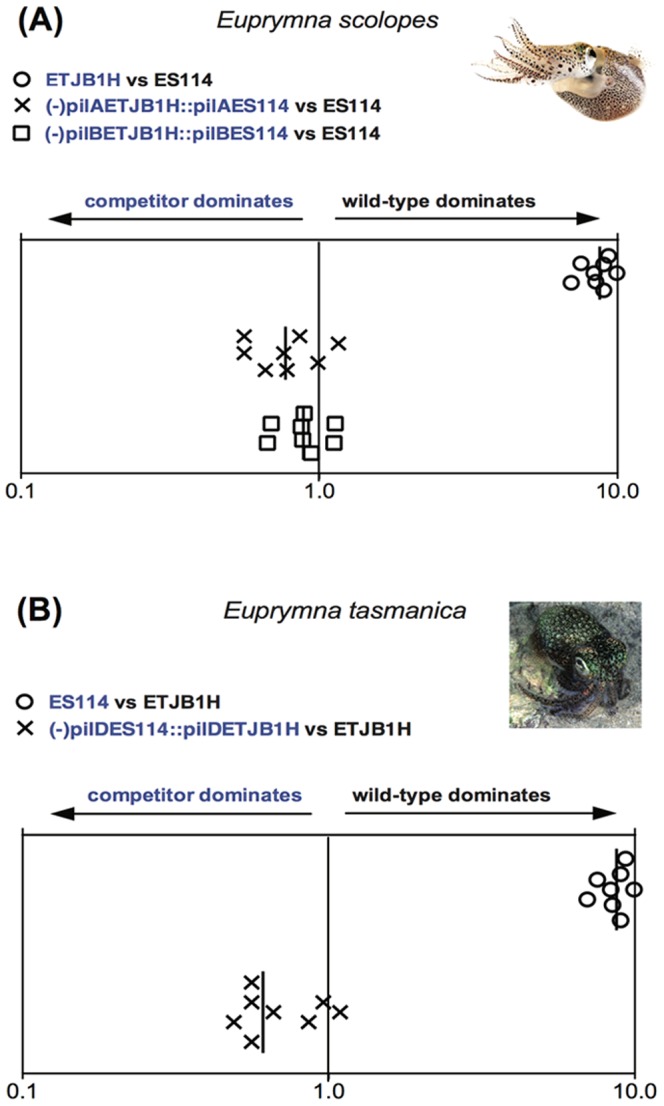
*pil* operon data. Colonization assays 48-hour post-infection of juvenile (A) *Euprymna scolopes* and (B) *Euprymna tasmanica* by their respective wild-type (ES114 or ETJB1H), mutant, and complement strains of *pil* genes for *Vibrio fischeri*. Infection efficiency data is plotted as the log values of the relative competitiveness index (RCIs), calculated by dividing the ratio of mutant to wild-type by the starting ratio [28]. If the RCI is <1 the mutant strain was outcompeted by the wild-type, the wild-type strain was outcompeted by the mutant if the value is >1, and a RCI equal to 1 indicates no competitive difference. Data points represent individual animals and the position of the figures on the y axis is merely for spacing. Vertical line represents the median value of each data plot.

We also created null *pil* mutants, and complemented them *in trans* to examine host selection. Colonization experiments in Hawaiian *E. scolopes* hatchlings indicate that *pilA, pilB* and *pilD* have important roles in host specificity (Figs. 3, S3). When constructs containing the native complemented gene were competed with either native or non-native wild-type strains, colonization efficiency of the constructed strains was equal or greater than the wild-type strain (Fig. 3). Similar results were observed in the case of *E. tasmanica* infection studies; however, *pilD* was the only locus that demonstrated host specificity in *E. tasmanica* (and not *pilA* or *pilB*). Genes from the *pil* operon (*pil ABCD*) encode for assembly of type IV pili, and are essential for bacterial attachment to both abiotic surfaces and to host cells [16], [19]. In *V. fischeri*, pilus subunits are synthesized by the *pilABCD* operon, where *pilA* contributes to colonization effectiveness and encodes a protein similar to type IV-A pilins (where *mshA* is a close relative [25], [26]. Phylogenetic and molecular differences have also been observed in *pilB* and *pilD* loci among multiple *V. fischeri* strains isolated from different squid hosts [27]. Our study demonstrates that *V. fischeri* ETJB1C *pilC* complemented with non-native Hawaiian *V. fischeri* ES114 *pilC* (-*pilC* ETJB1H::*pilC* ES114) is dominant in *E. scolopes*, but loses in *E. tasmanica*, since there is a competitive dominance for the native *pilC* locus (Fig. S3). Similar results are observed with *V. fischeri* ES114 *pilC* complemented with non-native Australian *V. fischeri* ETJB1H from *E. tasmanica*. This may be due to PilC being a phase variable protein (with minor differences in 3–5 aminoacids [25], which besides being implicated in type IV pilus biogenesis, mediates cell adherence [26]. Also, the heterogeneity of pili morphology means that multiple minor proteins composed of PilC subunits have evolved to be variable in order to compete for pilus receptors in host cells [25]. The intriguing question of how Pil-dependent binding is modulated and controlled between closely related host species may explain how host-switching can be accomplished through slight variations at this locus.

Additionally, we constructed complements containing the *native genes* and performed single colonization experiments as well as competition studies. Single colonization assays indicated that the complement was able to regain the colonization efficiency observed in the wild-type strain (data not shown), and the competition experiments (where the wild-type strain is used to co-infect the host with the native complement) indicated equivalent colonization efficiency between the two strains (Fig. S4).

To determine whether deletion of the various genetic elements had polar effects, we performed additional experiments to observe whether our mutants can affect phenotypes related to the function of downstream genes in the operon. For example, *msh* and *pil* influence adhesion and biofilm formation [21], and *lux* is responsible for light production [9]. We quantified biofilm and light production in both mutants and mutants complemented with the native gene, or in the case of *lux*, genes. Biofilm production decreased in mutant *msh* and *pil* strains, and light production was also impaired in *lux* mutants. Each phenotype was recovered in the respective native complements (data not shown), indicating that polarity effects may not be present; however, to be certain of this assumption additional studies are planned to determine if there is an influence in additional phenotypes. Future studies include transcriptional profiling and genetic analyses of metabolic pathways that might be affected in the various mutant strains.

Previous work examining experimental evolution of *V. fischeri* demonstrated polymorphic changes in phenotype (e.g., bioluminescence, biofilm, motility, growth) when strains are evolved in a novel host, allowing greater colonization efficiency of evolved strains when competed against their non-evolved ancestor [5], [24]. These results are consistent with our directionally mutated strains reported here, where non-native strains complemented with native loci (in both Hawaiian *E. scolopes* and Australian *E. tasmanica*) out-competed non-native strains and competed favorably over native parental strains during colonization in both host species examined. Our results indicate that the operons examined here are critical host-specificity factors and sufficient to dictate host recognition among closely related strains of *V. fischeri* from different geographical origins. Thus, strain specificity between two closely related *V. fischeri* symbionts from similar hosts is not mediated by a single or few loci, but rather multiple bacterial genetic elements that determine host range in allopatric Indo-west Pacific *Euprymna-Vibrio* associations.

Colonization of the squid host is multifactorial, and different studies from our laboratory have demonstrated that genes that are responsible for phenotypes associated to colonization are important for successful infection; additionally, experimental evolution of closely related strains does lead to competitive dominance of non-native strains [5], [24]. Although these studies suggest that these loci are important for host preference, there is the possibility that compensatory mechanisms could overtake the effect of a mutation and regulatory mechanisms (along with genetic factors) are responsible for colonization efficiency.

This study provides additional support of how bacterial diversity can be maintained through host selection, and key symbiotic loci are just one factor in determining host specificity. Determining whether these loci are acting in concert with one another to further push the selective advantage of beneficial vibrios is crucial for our understanding the evolution of symbiotic associations. How these subtle differences arise in wild populations, and whether they confer a greater selective advantage in bacterial fitness, will give insight into the processes of ecological adaptation in *Vibrio* bacteria.

## Supporting Information

Figure S1 **A**) Colonization assays 48-hour post-infection of juvenile *Euprymna scolopes* by wild-type, mutant, and complement strains of the *lux* operon for *Vibrio fischeri*. Single strain infection experiments are represented when only a single bar is shown (□). Competition experiments are represented with two bars (□ is the first strain and ▪ is the second strain), and each strain used is indicated below each competition. Wild-type ES114 significantly colonized the host better than non-native ETJB1H. Apo = aposymbiotic or non-infected juvenile squids. Data are plotted as the mean of Colony Forming Units (CFUs) counted for each strain. Multiple comparisons were calculated between groups using the Tukey PostHoc comparison. Different letters indicate significant differences (p<0.05) between groups or infection sets. See Table S1 for a complete description of strains and Table S2 for a complete description of colonization experiments. **B**) Colonization assays 48-hour post-infection of juvenile *Euprymna tasmanica* by wild-type, mutant, and complement strains of the *lux* operon for *Vibrio fischeri*. Single strain infection experiments are represented with a single bar (□). Competition experiments are represented with two bars (□ is the first strain and ▪ is the second strain), and each strain used is indicated below each competition. Wild-type Australian *V. fischeri* ETJB1H significantly colonized the host better than the non-native Hawaiian *V. fischeri* ES114. Apo = aposymbiotic or non-infected juvenile squids. Data are plotted as the mean of Colony Forming Units (CFUs) counted for each strain. Multiple comparisons were calculated between groups using the Tukey PostHoc comparison. Different letters indicate significant differences (p<0.05) between groups or infection sets.
(TIFF)

Figure S2 **A**) Colonization assays 48-hour post-infection of juvenile *Euprymna scolopes* by wild-type, mutant, and complement strains of *msh* genes for *Vibrio fischeri*. Single strain infection experiments are represented when only a single bar is shown (□). Competition experiments are represented with two bars (□ is the first strain and ▪ is the second strain), and each strain used is indicated below each competition. Wild-type ES114 significantly colonized its native host better than non-native ETJB1H. Apo = aposymbiotic or non-infected juvenile squids. Data are plotted as the mean of Colony Forming Units (CFUs) counted for each strain. Multiple comparisons were calculated between groups using the Tukey PostHoc comparison. Different letters indicate significant differences (p<0.05) between groups or infection sets. **B**) Colonization assays 48-hour post-infection of juvenile *Euprymna tasmanica* by wild-type, mutant, and complement strains of the *msh* genes for *Vibrio fischeri*. Single strain infection experiments are represented when only a single bar is shown (□). Competition experiments are represented with two bars (□ is the first strain and ▪ is the second strain), and each strain used is indicated below each competition. Wild-type ETJB1H significantly colonized the host better than the non-native ES114. Apo = aposymbiotic or non-infected juvenile squids. Data are plotted as the mean of Colony Forming Units (CFUs) counted for each strain. Multiple comparisons were calculated between groups using the Tukey PostHoc comparison. Different letters indicate significant differences (p<0.05) between groups or infection sets.
(TIFF)

Figure S3 **A**) Colonization assays 48-hour post-infection of juvenile *Euprymna scolopes* by wild-type, mutant, and complement strains of the *pil* genes for *Vibrio fischeri*. Single strain infection experiments are represented when only a single bar is shown (□). Competition experiments are represented with two bars (□ is the first strain and ▪ is the second strain), and each strain used is indicated below each competititon. Wild-type ES114 significantly colonized the host better than the non-native ETJB1H. Apo = aposymbiotic or non-infected juvenile squids. Data are plotted as the mean of Colony Forming Units (CFUs) counted for each strain. Multiple comparisons were calculated between groups using the Tukey PostHoc comparison. Different letters indicate significant differences (p<0.05) between groups or infection sets. **B**) Colonization assays 48-hour post-infection of juvenile *Euprymna tasmanica* by wild-type, mutant, and complement strains of *pil* genes for *Vibrio fischeri*. Single strain infection experiments are represented when only a single bar is shown (□). Competition experiments are represented with two bars (□ is the first strain and ▪ is the second strain), and each strain used is indicated below each competition. Wild-type ETJB1H significantly colonized the host better than the non-native ES114. Apo = aposymbiotic or non-infected juvenile squids. Data are plotted as the mean of Colony Forming Units (CFUs) counted for each strain. Multiple comparisons were calculated between groups using the Tukey PostHoc comparison. Different letters indicate significant differences (p<0.05) between groups or infection sets.
(TIFF)

Figure S4 **A**) Colonization assays 48-hour post-infection of juvenile *Euprymna scolopes* by wild-type and complement strains of the native genes for *Vibrio fischeri*. Single strain infection experiments are represented when only a single bar is shown (▪). Competition experiments are represented with two bars (▪ is the first strain and □ is the second strain). Data are plotted as the mean of Colony Forming Units (CFUs) counted for each strain. Multiple comparisons were calculated between groups using the one-way ANOVA test and Tukey PostHoc comparison. There was no significant difference between strains (as indicated by the *P* value of each column factor). **B**) Colonization assays 48-hour post-infection of juvenile *Euprymna tasmanica* by wild-type and complement strains of native genes for *Vibrio fischeri*. Single strain infection experiments are represented when only a single bar is shown (▪). Competition experiments are represented with two bars (▪ is the first strain and □ is the second strain). Data are plotted as the mean of Colony Forming Units (CFUs) counted for each strain. Multiple comparisons were calculated between groups using the one-way ANOVA test and Tukey PostHoc comparison. There was no significant difference between strains.
(TIF)

Table S1 Plasmids used and constructed in this study.
(DOCX)

Table S2 Strains used and constructed in this study.
(DOCX)
