# Supplementary figures and images for: Gene-Swapping Mediates Host Specificity among Symbiotic Bacteria in a Beneficial Symbiosis
Source: PLoS One. 2014 Jul 11;9(7):e101691. doi: 10.1371/journal.pone.0101691 (PMC4094467; doi:10.1371/journal.pone.0101691)

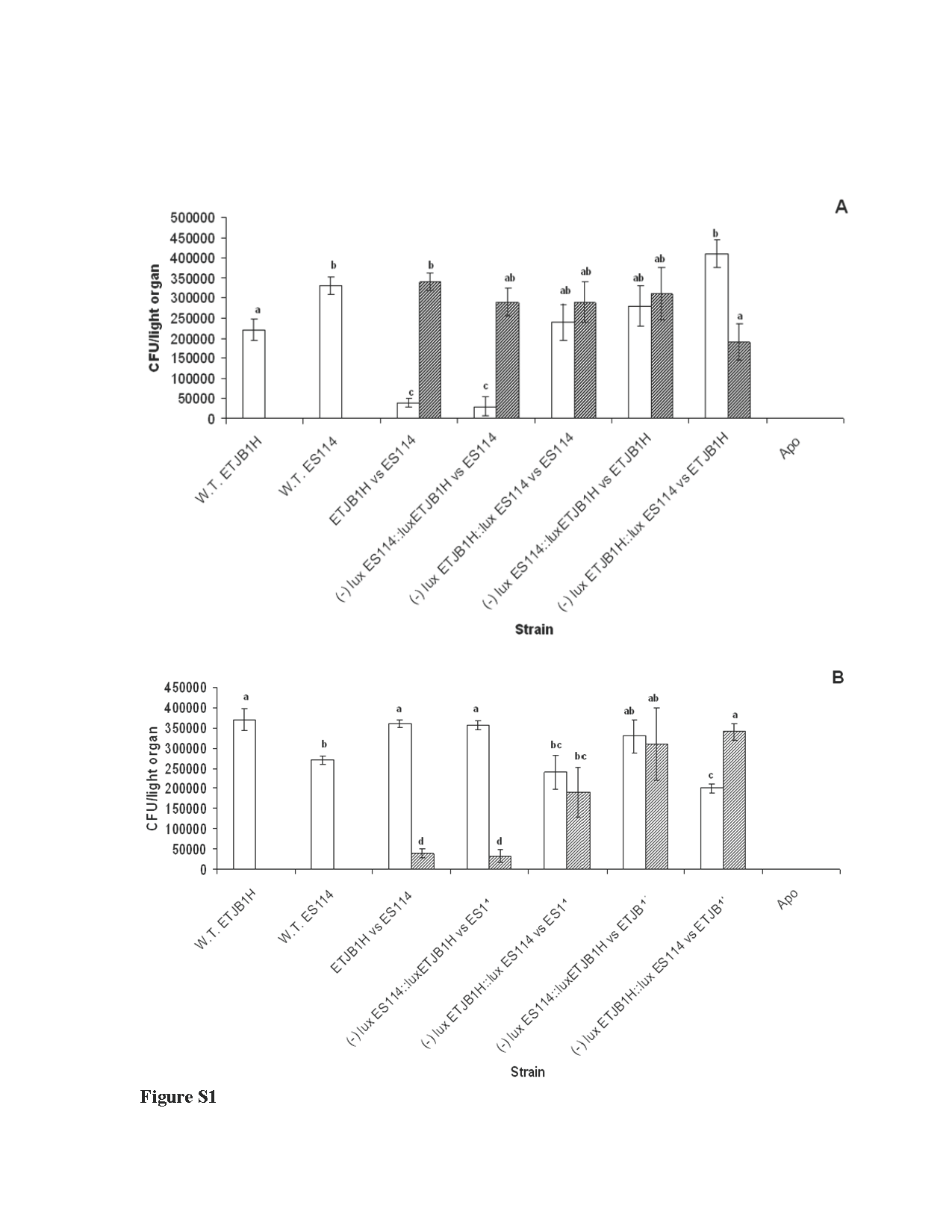

Supplement: Figure S1 — A) Colonization assays 48-hour post-infection of juvenile Euprymna scolopes by wild-type, mutant, and complement strains of the lux operon for Vibrio fischeri. Single strain infection experiments are represented when only a single bar is shown (□). Competition experiments are represented with two bars (□ is the first strain and ▪ is the second strain), and each strain used is indicated below each competition. Wild-type ES114 significantly colonized the host better than non-native ETJB1H. Apo = aposymbiotic or non-infected juvenile squids. Data are plotted as the mean of Colony Forming Units (CFUs) counted for each strain. Multiple comparisons were calculated between groups using the Tukey PostHoc comparison. Different letters indicate significant differences (p<0.05) between groups or infection sets. See Table S1 for a complete description of strains and Table S2 for a complete description of colonization experiments. B) Colonization assays 48-hour post-infection of juvenile Euprymna tasmanica by wild-type, mutant, and complement strains of the lux operon for Vibrio fischeri. Single strain infection experiments are represented with a single bar (□). Competition experiments are represented with two bars (□ is the first strain and ▪ is the second strain), and each strain used is indicated below each competition. Wild-type Australian V. fischeri ETJB1H significantly colonized the host better than the non-native Hawaiian V. fischeri ES114. Apo = aposymbiotic or non-infected juvenile squids. Data are plotted as the mean of Colony Forming Units (CFUs) counted for each strain. Multiple comparisons were calculated between groups using the Tukey PostHoc comparison. Different letters indicate significant differences (p<0.05) between groups or infection sets. (TIFF) [file pone.0101691.s001.tiff]

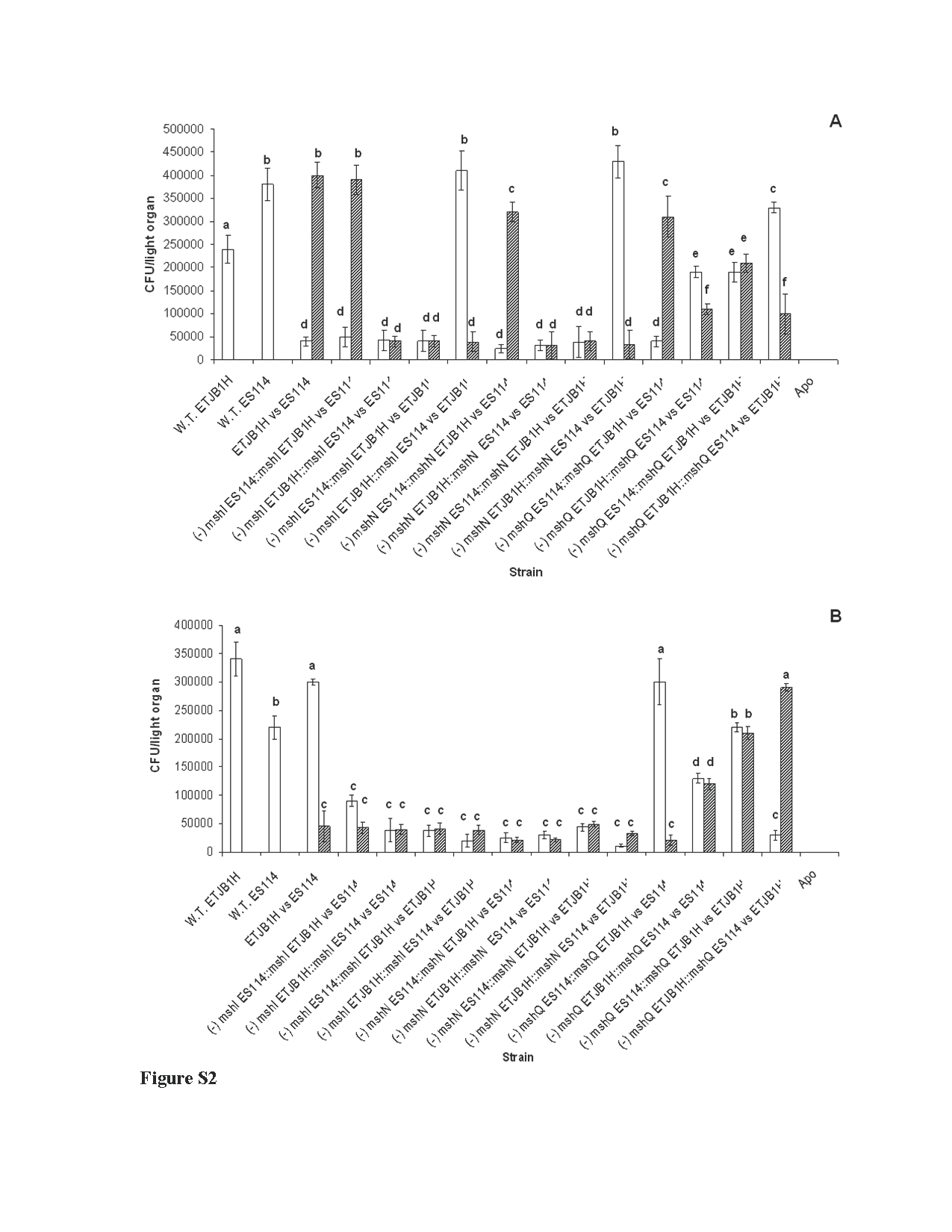

Supplement: Figure S2 — A) Colonization assays 48-hour post-infection of juvenile Euprymna scolopes by wild-type, mutant, and complement strains of msh genes for Vibrio fischeri. Single strain infection experiments are represented when only a single bar is shown (□). Competition experiments are represented with two bars (□ is the first strain and ▪ is the second strain), and each strain used is indicated below each competition. Wild-type ES114 significantly colonized its native host better than non-native ETJB1H. Apo = aposymbiotic or non-infected juvenile squids. Data are plotted as the mean of Colony Forming Units (CFUs) counted for each strain. Multiple comparisons were calculated between groups using the Tukey PostHoc comparison. Different letters indicate significant differences (p<0.05) between groups or infection sets. B) Colonization assays 48-hour post-infection of juvenile Euprymna tasmanica by wild-type, mutant, and complement strains of the msh genes for Vibrio fischeri. Single strain infection experiments are represented when only a single bar is shown (□). Competition experiments are represented with two bars (□ is the first strain and ▪ is the second strain), and each strain used is indicated below each competition. Wild-type ETJB1H significantly colonized the host better than the non-native ES114. Apo = aposymbiotic or non-infected juvenile squids. Data are plotted as the mean of Colony Forming Units (CFUs) counted for each strain. Multiple comparisons were calculated between groups using the Tukey PostHoc comparison. Different letters indicate significant differences (p<0.05) between groups or infection sets. (TIFF) [file pone.0101691.s002.tiff]

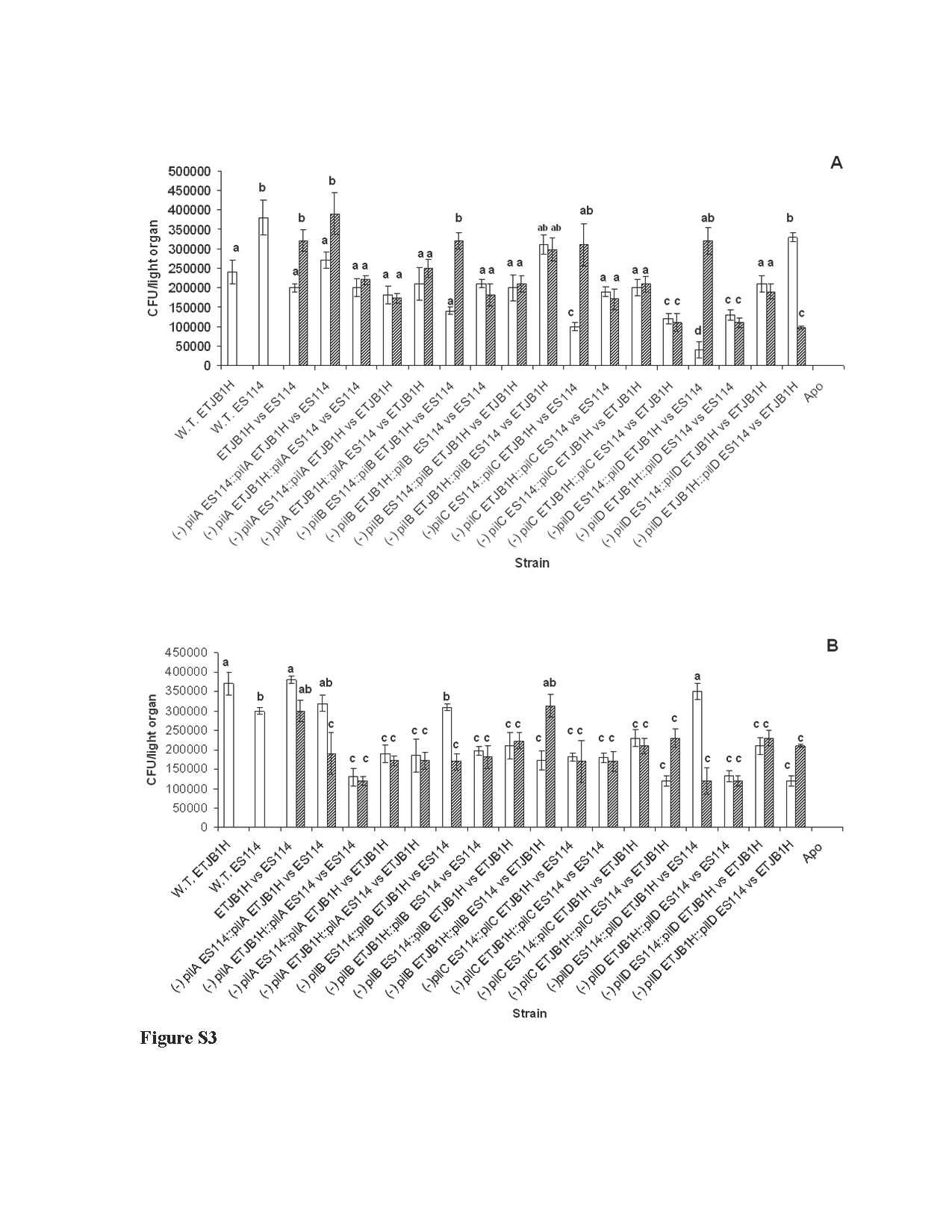

Supplement: Figure S3 — A) Colonization assays 48-hour post-infection of juvenile Euprymna scolopes by wild-type, mutant, and complement strains of the pil genes for Vibrio fischeri. Single strain infection experiments are represented when only a single bar is shown (□). Competition experiments are represented with two bars (□ is the first strain and ▪ is the second strain), and each strain used is indicated below each competititon. Wild-type ES114 significantly colonized the host better than the non-native ETJB1H. Apo = aposymbiotic or non-infected juvenile squids. Data are plotted as the mean of Colony Forming Units (CFUs) counted for each strain. Multiple comparisons were calculated between groups using the Tukey PostHoc comparison. Different letters indicate significant differences (p<0.05) between groups or infection sets. B) Colonization assays 48-hour post-infection of juvenile Euprymna tasmanica by wild-type, mutant, and complement strains of pil genes for Vibrio fischeri. Single strain infection experiments are represented when only a single bar is shown (□). Competition experiments are represented with two bars (□ is the first strain and ▪ is the second strain), and each strain used is indicated below each competition. Wild-type ETJB1H significantly colonized the host better than the non-native ES114. Apo = aposymbiotic or non-infected juvenile squids. Data are plotted as the mean of Colony Forming Units (CFUs) counted for each strain. Multiple comparisons were calculated between groups using the Tukey PostHoc comparison. Different letters indicate significant differences (p<0.05) between groups or infection sets. (TIFF) [file pone.0101691.s003.tiff]

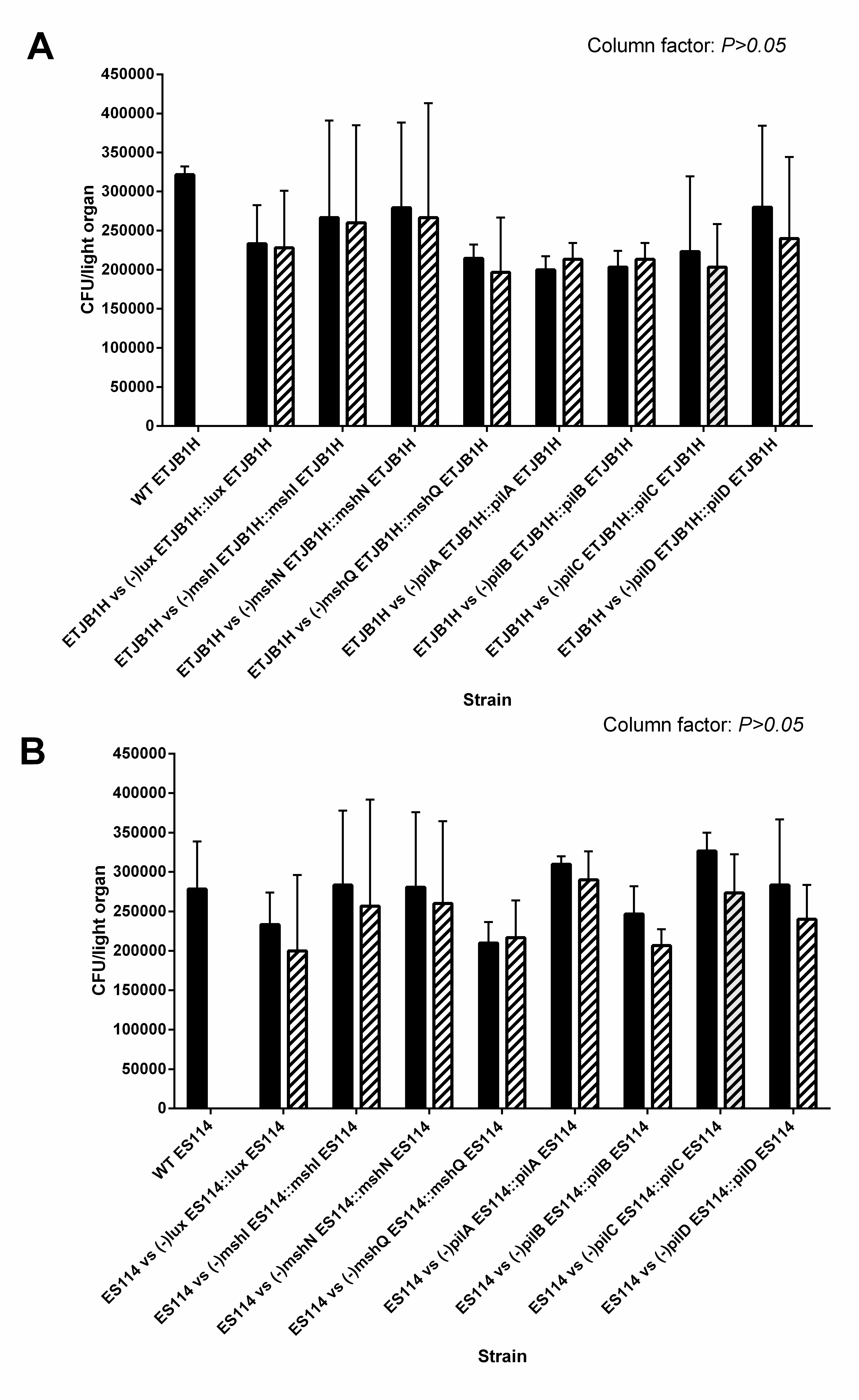

Supplement: Figure S4 — A) Colonization assays 48-hour post-infection of juvenile Euprymna scolopes by wild-type and complement strains of the native genes for Vibrio fischeri. Single strain infection experiments are represented when only a single bar is shown (▪). Competition experiments are represented with two bars (▪ is the first strain and □ is the second strain). Data are plotted as the mean of Colony Forming Units (CFUs) counted for each strain. Multiple comparisons were calculated between groups using the one-way ANOVA test and Tukey PostHoc comparison. There was no significant difference between strains (as indicated by the P value of each column factor). B) Colonization assays 48-hour post-infection of juvenile Euprymna tasmanica by wild-type and complement strains of native genes for Vibrio fischeri. Single strain infection experiments are represented when only a single bar is shown (▪). Competition experiments are represented with two bars (▪ is the first strain and □ is the second strain). Data are plotted as the mean of Colony Forming Units (CFUs) counted for each strain. Multiple comparisons were calculated between groups using the one-way ANOVA test and Tukey PostHoc comparison. There was no significant difference between strains. (TIF) [file pone.0101691.s004.tif]
