# Supplementary material for: Gene-Swapping Mediates Host Specificity among Symbiotic Bacteria in a Beneficial Symbiosis
Source: PLoS One. 2014 Jul 11;9(7):e101691. doi: 10.1371/journal.pone.0101691 (PMC4094467; doi:10.1371/journal.pone.0101691)
Supplement: Table S1 — Plasmids used and constructed in this study. (DOCX) [file pone.0101691.s005.docx]

**Table S1.** Plasmids used and constructed in this study.

| **Plasmid** | **Genotypes or markers** |
| --- | --- |
| pEVS122 | R6Kγ OriV *lacZα* ^a^Erm^R^ |
| pVSV105 | pES213 replicon, R6Kγ OriV *lacZα* ^a^Cm^R^ |
| pCRG13 | CYH2 OricolE1 ^a^Amp^R^ |
| pCRG23 | URA3 OriF’ ^a^Cm^R^ |
| pSW7848 | R6Kγ OriV RP4 OriT::*araC*-P_BAD_*^ccdB^* |
| ***lux* constructs** | |

resistance) ^b^NCBI locus tag
