# Supplementary material for: Gene-Swapping Mediates Host Specificity among Symbiotic Bacteria in a Beneficial Symbiosis
Source: PLoS One. 2014 Jul 11;9(7):e101691. doi: 10.1371/journal.pone.0101691 (PMC4094467; doi:10.1371/journal.pone.0101691)
Supplement: Table S2 — Strains used and constructed in this study. (DOCX) [file pone.0101691.s006.docx]

**Table S2.** Strains used and constructed in this study.

| **Strains** | **Description** |
| --- | --- |
| ***S. cerevisiae* strains** | |
| CRY 1-2 | ^a^cyh2^R^, ura^-^ |
| TYC1^2^ | ^a^cyh2^R^, ura^-^, leu^-^, ∆*lig*4 |
| ***Vibrio fischeri* strains** | |
| ETJB1H | Wild-type from *Eupyrmna tasmanica*, Jervis Bay, New South Wales, Australia |
| ES114 | Wild-type from *Euprymna scolopes*, Kane’ohe Bay, Hawaii |
| *Mutants* | |
| ES114 pEVS122::*luxA* | ES114::pACH101 |
| ETJB1H pEVS122::*luxA* | ETJB1H::pACH102 |
| ES114 ∆*mshI* | ES114::pACH104 |
| ES114 ∆*mshN* | ES114::pACH108 |
| ES114 ∆*mshQ* | ES114::pACH112 |
| ETJB1H ∆*mshI* | ETJB1H::pACH104 |
| ETJB1H ∆*mshN* | ETJB1H::pACH108 |
| ETJB1H ∆*mshQ* | ETJB1H::pACH112 |
| ES114 ∆*pilA* | ES114::pACH116 |
| ES114 ∆*pilB* | ES114::pACH120 |
| ES114 ∆*pilC* | ES114::pACH124 |
| ES114 ∆*pilD* | ES114::pACH128 |
| ETJB1H ∆*pilA* | ETJB1H::pACH116 |
| ETJB1H ∆*pilB* | ETJB1H::pACH120 |
| ETJB1H ∆*pilC* | ETJB1H::pACH124 |
| ETJB1H ∆*pilD* | ETJB1H::pACH128 |
| *Complements* | |
| Comp ES114 pEVS122::*luxA* | ES114 pEVS122::*luxA*::pACH132 |
| Comp ETJB1H pEVS122::*luxA* | ETJB1H *pEVS122*::*luxA*::pACH131 |
| Comp ES114 ∆*mshI* | ES114 ∆*mshI*::pACH106 |
| Comp ES114 ∆*mshN* | ES114 ∆*mshN*::pACH110 |
| Comp ES114 ∆*mshQ* | ES114 ∆*mshQ*::pACH114 |
| Comp ETJB1H ∆*mshI* | ETJB1H ∆*mshI*::pACH105 |
| Comp ETJB1H ∆*mshN* | ETJB1H ∆*mshN*::pACH109 |
| Comp ETJB1H ∆*mshQ* | ETJB1H ∆*mshQ*::pACH113 |
| Comp ES114 ∆*pilA* | ES114 ∆*pilA*::pACH118 |
| Comp ES114 ∆*pilB* | ES114 ∆*pilB*::pACH122 |
| Comp ES114 ∆*pilC* | ES114 ∆*pilC*::pACH126 |
| Comp ES114 ∆*pilD* | ES114 ∆*pilD*::pACH130 |
| Comp ETJB1H ∆*pilA* | ES114 ∆*pilA*::pACH117 |
| Comp ETJB1H ∆*pilB* | ES114 ∆*pilB*::pACH121 |
| Comp ETJB1H ∆*pilC* | ES114 ∆*pilC*::pACH125 |
| Comp ETJB1H ∆*pilD*  Comp native ES114 *luxA*  Comp native ETJB1H *luxA*  Comp native ES114 ∆*mshI*  Comp native ES114∆*mshN*  Comp native ES114∆*mshQ*  Comp native ETJB1H∆*mshI*  Comp native ETJB1H∆*mshN*  Comp nativeETJB1H∆*mshQ*  Comp native ES114∆*pilA*  Comp native ES114∆*pilB*  Comp native ES114∆*pilC*  Comp native ES114∆*pilD*  Comp native ETJB1H∆*pilA*  Comp native ETJB1H∆*pilB*  Comp native ETJB1H∆*pilC*  Comp native ETJB1H∆*pilD* | ES114 ∆*pilD*::pACH129  ES114 pEVS122::*luxA::*pACH101  ES114 pEVS122::*luxA*::pACH102  ES114∆*mshI*::pACH105  ES114∆*mshN*::pACH109  ES114∆*mshQ*::pACH113  ETJB1H∆*mshI*::pACH106  ETJB1H∆*mshN*::pACH110  ETJB1H∆*mshQ*::pACH114  ES114∆*pilA*::pACH117  ES114∆*pilB*::pACH121  ES114∆*pilC*::pACH125  ES114∆*pilD*::pACH129  ETJB1H∆*pilA*::pACH118  ETJB1H∆*pilB*::pACH122  ETJB1H∆*pilC*::pACH126  ETJB1H∆*pilD::*pACH130 |

^a^cyh2^R^(Cyclohexemide resistance)
